# Supplementary material for: The effective connectome over a century of human life
Source: Commun Biol. 2025 Nov 24;8:1638. doi: 10.1038/s42003-025-08970-4 (PMC12644845; doi:10.1038/s42003-025-08970-4)
Supplement: Supplementary file 2 — Supplemental Information [file 42003_2025_8970_MOESM2_ESM.pdf]

Supplemental Information

Guoshi Li[1,2], Khoi Minh Huynh[1,2], Kim-Han Thung[1,2], Hoyt Patrick Taylor IV[1,2], Guoye Lin[1,2], Weili Lin[1,2], Sahar Ahmad[1,2], & Pew-Thian Yap[1,2,✉]

[1]*Department of Radiology, University of North Carolina at Chapel Hill, Chapel Hill, NC, USA*
[2]*Biomedical Research Imaging Center, University of North Carolina at Chapel Hill, Chapel Hill, NC, USA*

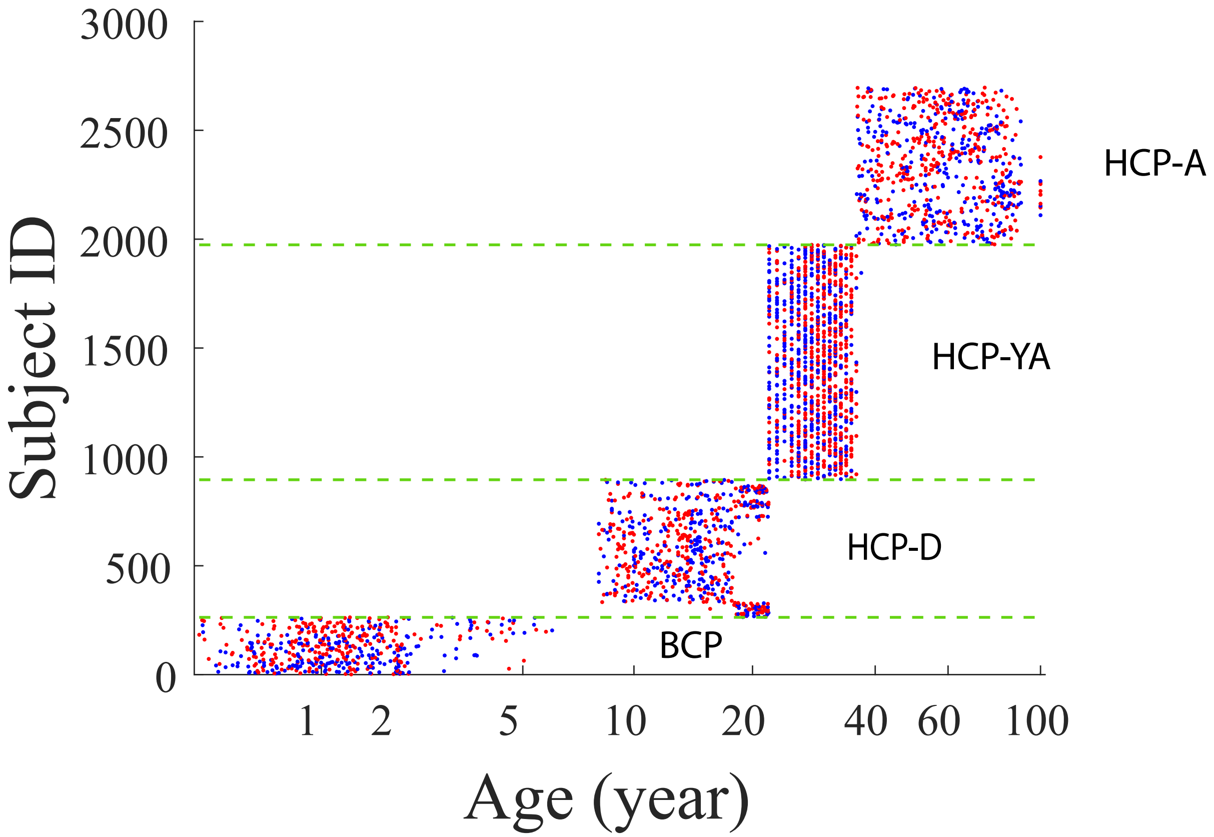

Figure S1 | Distribution of the scan age for all 2696 subjects. Males are shown in blue and females are shown in red.

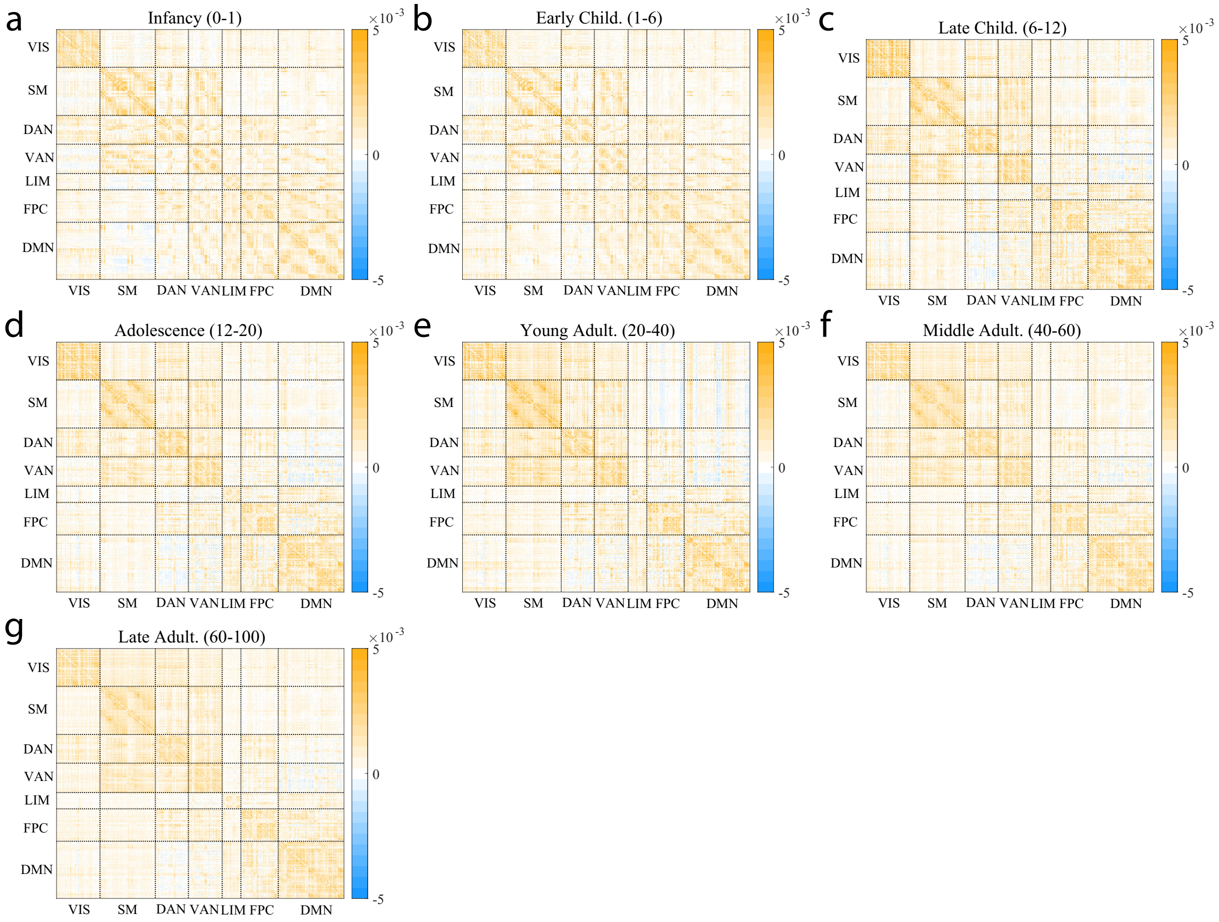

Figure S2 | Average effective connectivity (EC) in different developmental stages. **a**, Average EC in infancy (0 – 1 year). **b**, Average EC in early childhood (1 – 6 years). **c**, Average EC in late childhood (6 – 12 years). **d**, Average EC in adolescence (12 – 20 years). **e**, Average EC in young adulthood (20 – 40 years). **f**, Average EC in middle adulthood (40 – 60 years). **g**, Average EC in late adulthood (60 – 100 years). Note the diagonal component is removed in EC due to its large negative value. VIS: visual network, SM: somatomotor network, DAN: dorsal attention network, VAN: ventral attention network, LIM: limbic network, FPC: frontoparietal control network, DMN: default mode network.

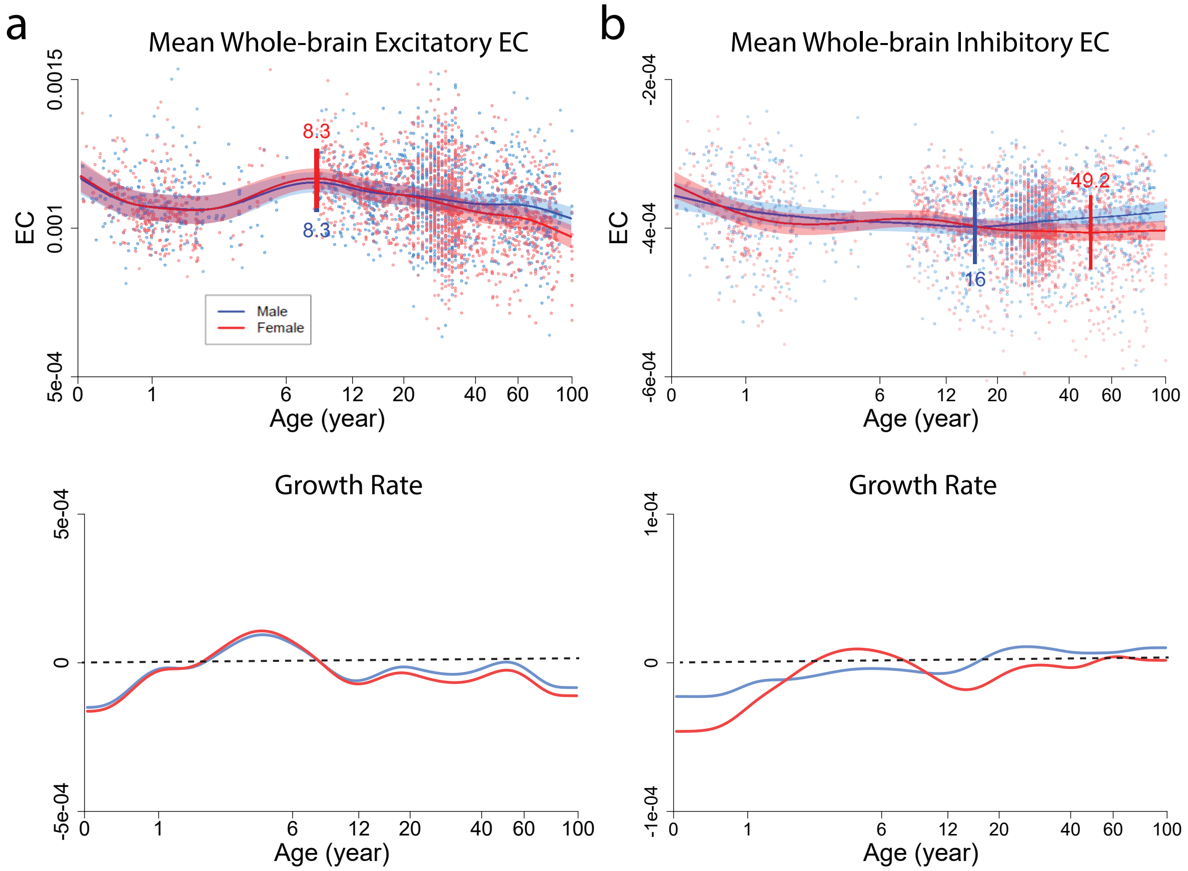

Figure S3 | **Evolution of the global excitatory and inhibitory EC over the lifespan.** **a**, Normative trajectories (*top*) and velocity curves (*bottom*) of the mean whole-brain excitatory EC. **b**, Normative trajectories (*top*) and velocity curves (*bottom*) of the mean whole-brain inhibitory EC. Background points denote EC metrics of individual subjects as a function of age; points are colored by sex. The horizontal axis is in log scale and the short vertical bars indicate the peak ages.

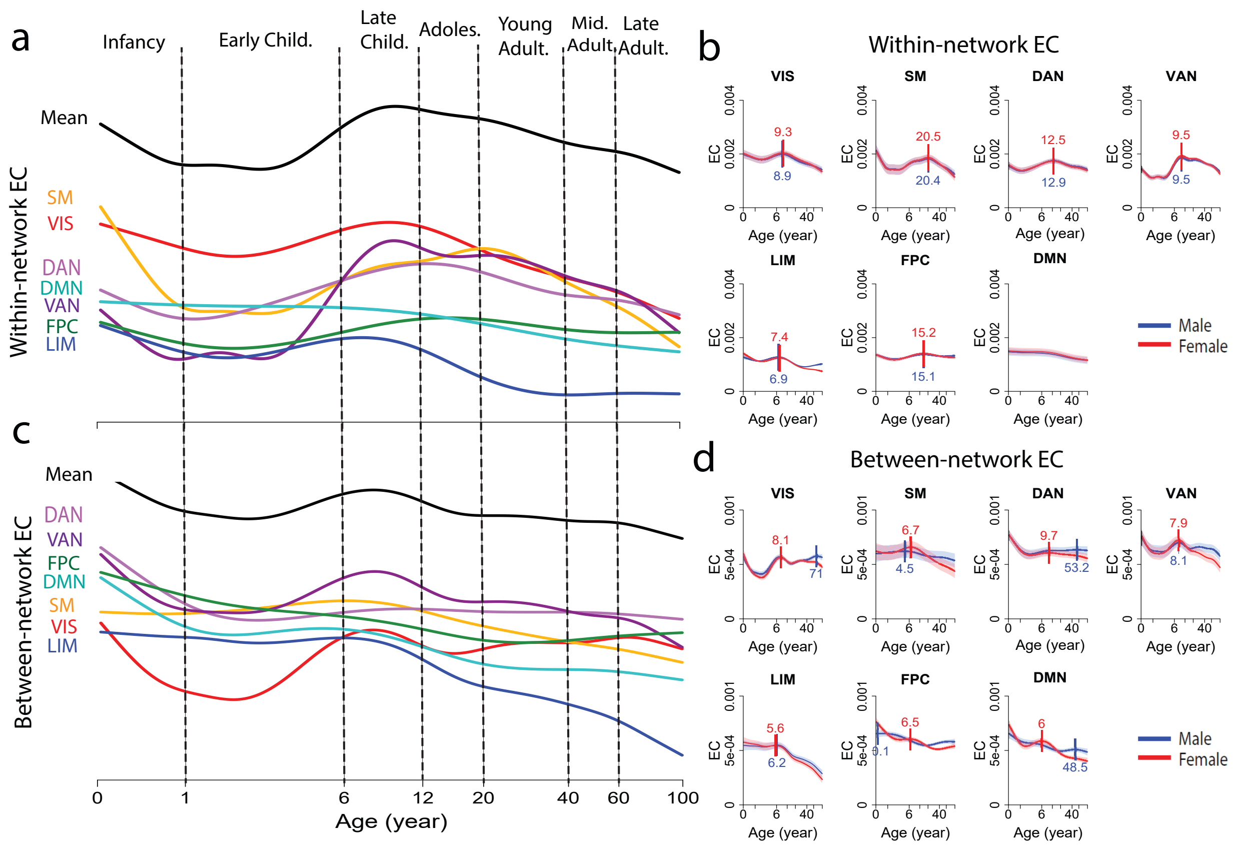

Figure S4 | **Evolution of within-network and between-network EC over the lifespan. a**, Developmental trajectories of the mean and network-specific within-network EC. **b**, Normative trajectories of within-network EC for each individual network stratified by sex. **c**, Developmental trajectories of the mean and network-specific between-network EC. **d**, Normative trajectories of between-network EC for each individual network stratified by sex. For a and c, the mean trajectory is manually lifted for better visualization. For b and d, the short vertical bars indicate the peak ages. For a–d, the horizontal axis is in log scale. VIS: visual network, SM: somatomotor network, DAN: dorsal attention network, VAN: ventral attention network, LIM: limbic network, FPC: frontoparietal control network, DMN: default mode network.

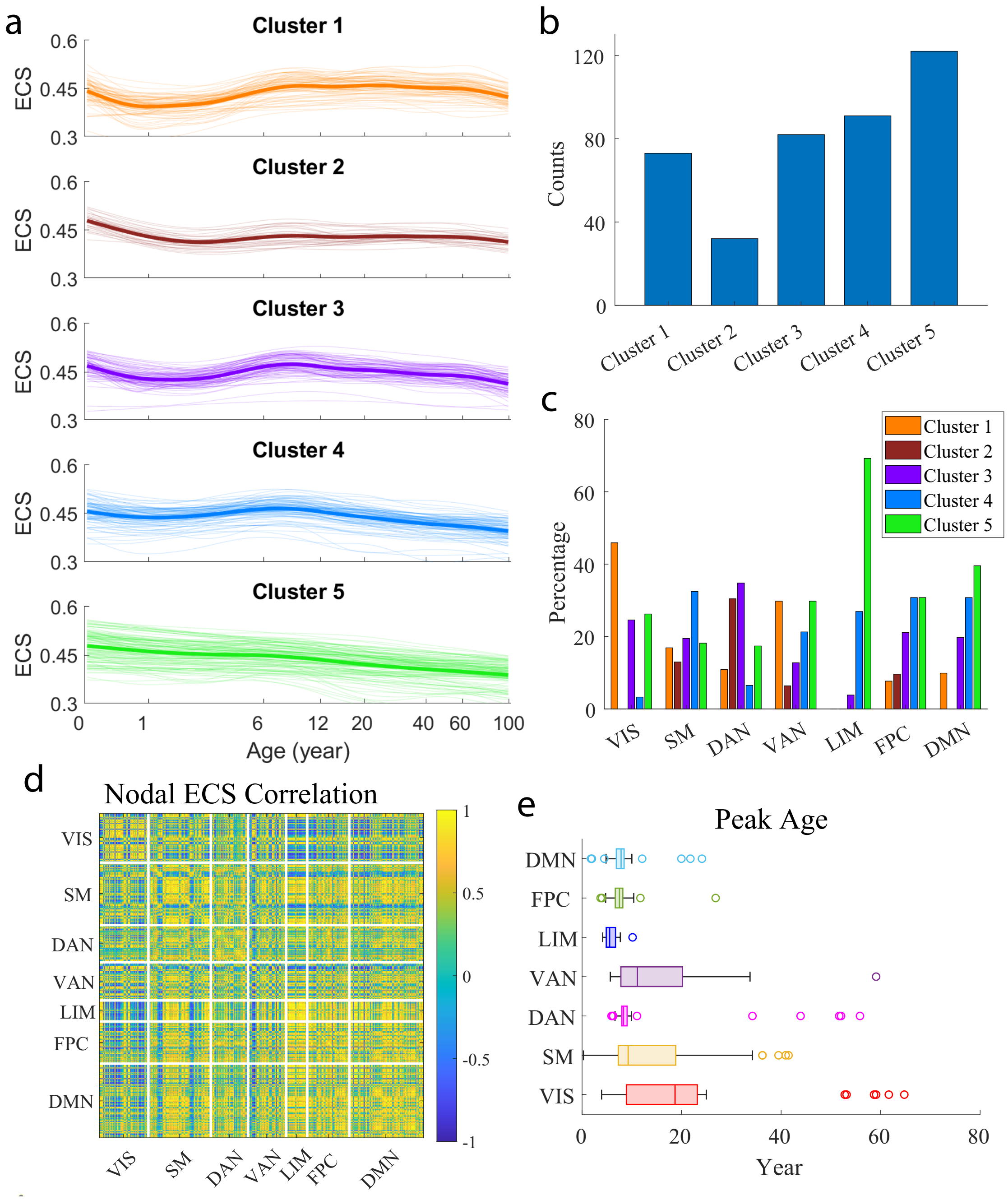

Figure S5 | Developmental patterns of excitatory nodal effective connectivity strength (ECS). **a**, Classification of 400 fitted nodal ECS time courses into five different clusters representting distinct temporal patterns. The average nodal ECS profile for each cluster is in bold shape. **b**, Distribution of the five clusters among 400 regions. **c**, Percentage of each cluster in each functional network. **d**, Correlation matrix of the 400 nodal ECS time series. **e**, Distribution of the peak ages of nodal ECS in each functional network. For the boxplots, the central mark indicates the median, and the left and right edges of the box indicate the 25th and 75th percentiles, respectively. The whiskers extend to the most extreme data points not considered outliers, and the outliers are plotted individually using the "o" marker symbol. For a, the horizontal axis is in log scale. VIS: visual network, SM: somatomotor network, DAN: dorsal attention network, VAN: ventral attention network, LIM: limbic network, FPC: frontoparietal control network, DMN: default mode network.

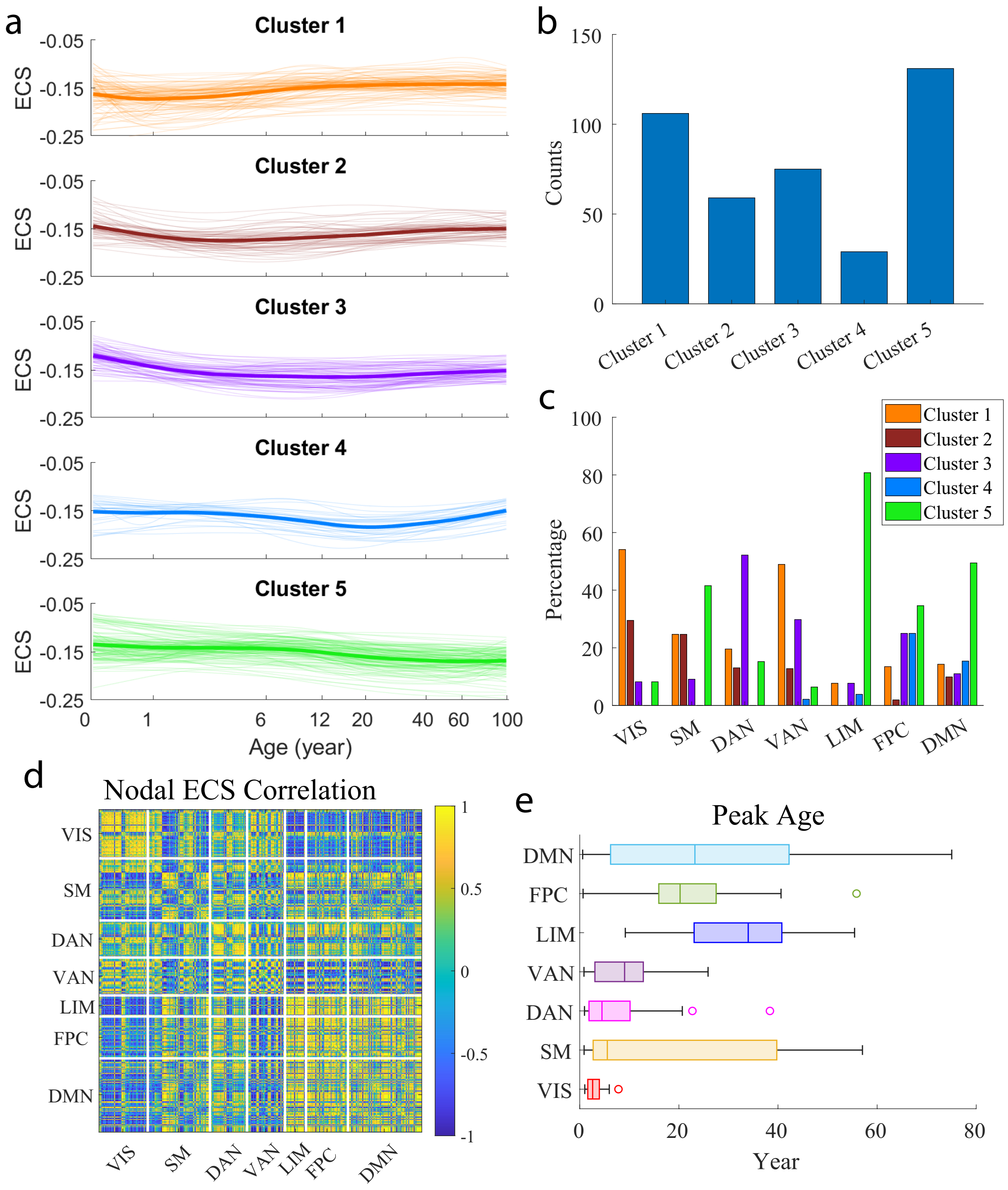

Figure S6 | **Developmental patterns of inhibitory nodal effective connectivity strength (ECS).** **a**, Classification of 400 fitted nodal ECS time courses into five different clusters representting distinct temporal patterns. The average nodal ECS profile for each cluster is in bold shape. **b**, Distribution of the five clusters among 400 regions. **c**, Percentage of each cluster in each functional network. **d**, Correlation matrix of the 400 nodal ECS time series. **e**, Distribution of the peak ages of nodal ECS in each functional network. For the boxplots, the central mark indicates the median, and the left and right edges of the box indicate the 25th and 75th percentiles, respectively. The whiskers extend to the most extreme data points not considered outliers, and the outliers are plotted individually using the "o" marker symbol. For a, the horizontal axis is in log scale. VIS: visual network, SM: somatomotor network, DAN: dorsal attention network, VAN: ventral attention network, LIM: limbic network, FPC: frontoparietal control network, DMN: default mode network.

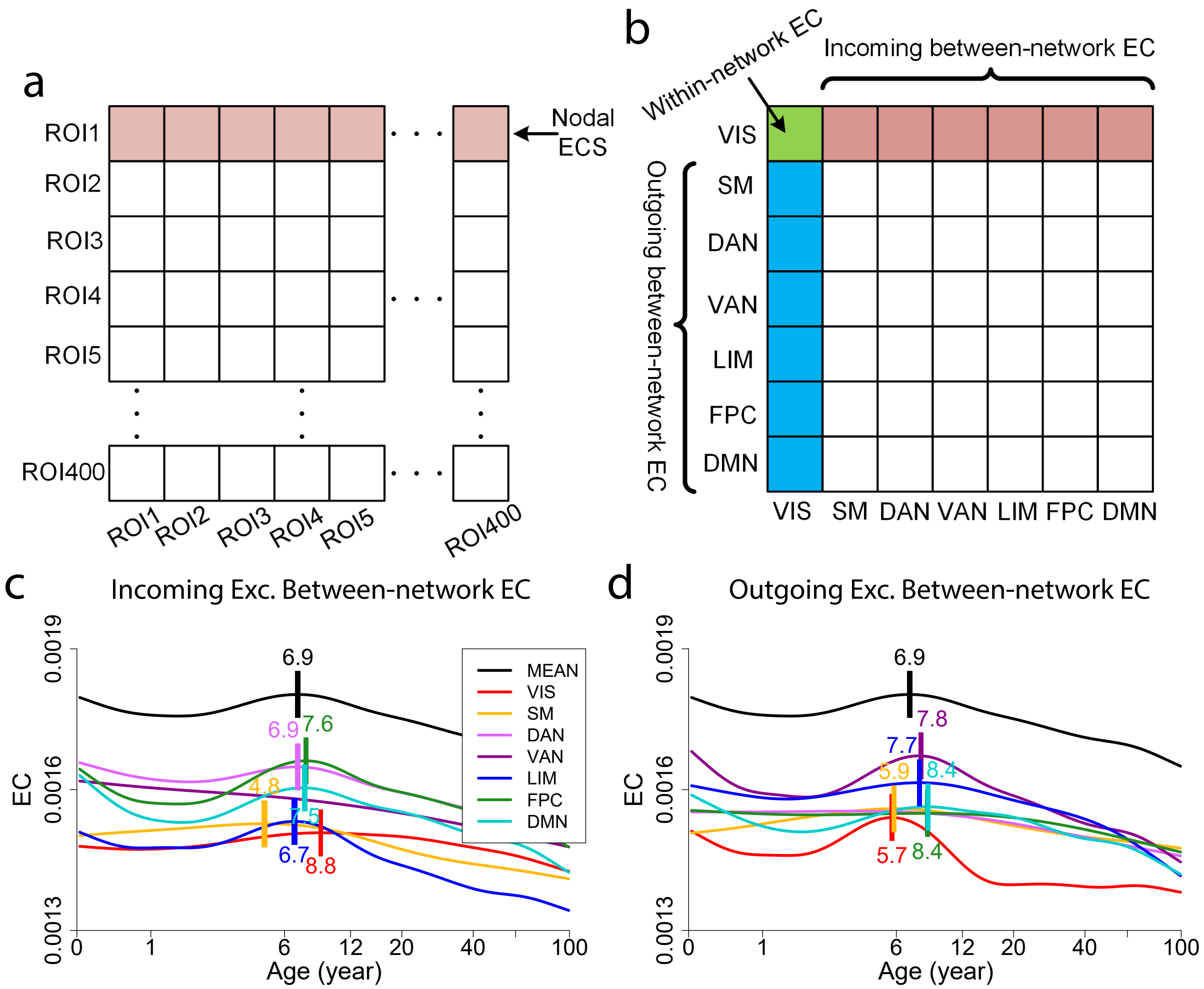

Figure S7 | **Differential peak ages for incoming and outgoing between-network EC. a**, Diagram illustrating nodal effective connectivity strength (ECS) which is the sum of all incoming EC to a particular region. **b**, Diagram illustrating within-network EC, incoming between-network EC and outgoing between-network EC for the visual network. **c**, Developmental trajectories of the mean and network-specific incoming excitatory between-network EC. **d**, Developmental trajectories of the mean and network-specific outgoing excitatory between-network EC. For c and d, the mean trajectory is manually lifted for better visualization, the horizontal axis is in log scale, and the short vertical bars indicate the peak ages. VIS: visual network, SM: somatomotor network, DAN: dorsal attention network, VAN: ventral attention network, LIM: limbic network, FPC: frontoparietal control network, DMN: default mode network.

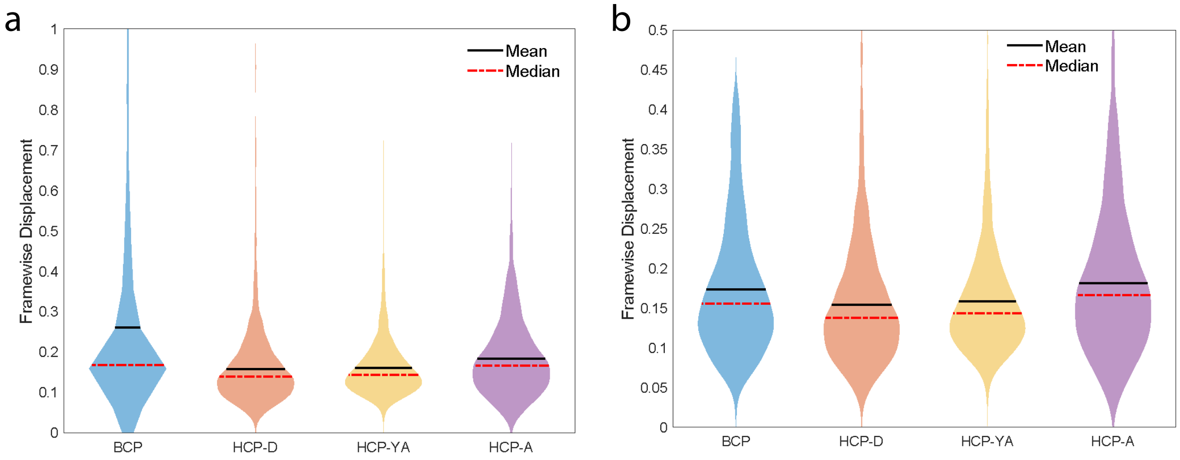

Figure S8 | Framewise displacement (FD) of HCP lifespan datasets. a, Violin plot of FD before quality control. **b**, Violin plot of FD after quality control.
