# Supplementary material for: The effective connectome over a century of human life
Source: Commun Biol. 2025 Nov 24;8:1638. doi: 10.1038/s42003-025-08970-4 (PMC12644845; doi:10.1038/s42003-025-08970-4)
Supplement: Supplementary file 3 — Description of Additional Supplementary files [file 42003_2025_8970_MOESM3_ESM.pdf]

Description of Additional Supplementary files

File name: Supplementary Data 1

Description: The source data for Figure 1

File name: Supplementary Data 2

Description: The source data for Figure 2.

File name: Supplementary Data 3

Description: The source data for Figure 3.

File name: Supplementary Data 4

Description: The source data for Figure 4.

File name: Supplementary Data 5

Description: The source data for Figure 5.
