# Supplementary material for: The effective connectome over a century of human life
Source: Commun Biol. 2025 Nov 24;8:1638. doi: 10.1038/s42003-025-08970-4 (PMC12644845; doi:10.1038/s42003-025-08970-4)
Supplement: Supplementary file 9 — Reporting Summary [file 42003_2025_8970_MOESM9_ESM.pdf]

nature portfolio

Corresponding author(s): Pew-Thian Yap

Last updated by author(s): Sep 12, 2025

Reporting Summary

Nature Portfolio wishes to improve the reproducibility of the work that we publish. This form provides structure for consistency and transparency in reporting. For further information on Nature Portfolio policies, see our Editorial Policies and the Editorial Policy Checklist.

Statistics

For all statistical analyses, confirm that the following items are present in the figure legend, table legend, main text, or Methods section.

n/a	Confirmed	
☐	☒	The exact sample size (*n*) for each experimental group/condition, given as a discrete number and unit of measurement
☐	☒	A statement on whether measurements were taken from distinct samples or whether the same sample was measured repeatedly
☐	☒	The statistical test(s) used AND whether they are one- or two-sided<br>*Only common tests should be described solely by name; describe more complex techniques in the Methods section.*
☐	☒	A description of all covariates tested
☐	☒	A description of any assumptions or corrections, such as tests of normality and adjustment for multiple comparisons
☐	☒	A full description of the statistical parameters including central tendency (e.g. means) or other basic estimates (e.g. regression coefficient) AND variation (e.g. standard deviation) or associated estimates of uncertainty (e.g. confidence intervals)
☐	☒	For null hypothesis testing, the test statistic (e.g. F, t, r) with confidence intervals, effect sizes, degrees of freedom and P value noted<br>*Give P values as exact values whenever suitable.*
☒	☐	For Bayesian analysis, information on the choice of priors and Markov chain Monte Carlo settings
☒	☐	For hierarchical and complex designs, identification of the appropriate level for tests and full reporting of outcomes
☐	☒	Estimates of effect sizes (e.g. Cohen's *d*, Pearson's *r*), indicating how they were calculated

Our web collection on statistics for biologists contains articles on many of the points above.

Software and code

Policy information about availability of computer code

Data collection	N/A. This study does not collect new data.
Data analysis	MATLAB (2024b) and R (2023.12)

For manuscripts utilizing custom algorithms or software that are central to the research but not yet described in published literature, software must be made available to editors and reviewers. We strongly encourage code deposition in a community repository (e.g. GitHub). See the Nature Portfolio guidelines for submitting code & software for further information.

Data

Policy information about availability of data

All manuscripts must include a data availability statement. This statement should provide the following information, where applicable:

- Accession codes, unique identifiers, or web links for publicly available datasets
- A description of any restrictions on data availability
- For clinical datasets or third party data, please ensure that the statement adheres to our policy

The Lifespan HCP fMRI data are publicly available via the National Institute of Mental Health data archive (NDA, http://nda.nih.gov)

Research involving human participants, their data, or biological material

Policy information about studies with human participants or human data. See also policy information about sex, gender (identity/presentation), and sexual orientation and race, ethnicity and racism.

Reporting on sex and gender	We included sex as a covariate in our generalized additive mixed model (GAMM). We have reported differences between males and females in the manuscript.
Reporting on race, ethnicity, or other socially relevant groupings	N/A
Population characteristics	We included 2,696 subjects (male/female: 1232/1464; age: 10 days to 100 years) from the Lifespan HCP studies. Specifically, we included 263 subjects (471 longitudinal scans; M/F: 124/139; age: 1.4 ± 1.0 year) from BCP, 632 subjects from HCP-Development (M/F: 294/338; age: 14.7 ± 3.9 year), 1079 subjects from HCP-Young Adult (M/F: 495/584; age: 28.8 ± 3.7 year), and 722 subjects from HCP-Aging (M/F: 319/403; age: 60.3 ± 15.7 year).
Recruitment	Data were not collected in this study.
Ethics oversight	Written informed consent of participants (or their guardians) was approved by the local ethics committees for each dataset.

Note that full information on the approval of the study protocol must also be provided in the manuscript.

Field-specific reporting

Please select the one below that is the best fit for your research. If you are not sure, read the appropriate sections before making your selection.

☒ Life sciences ☐ Behavioural & social sciences ☐ Ecological, evolutionary & environmental sciences

For a reference copy of the document with all sections, see nature.com/documents/nr-reporting-summary-flat.pdf

Life sciences study design

All studies must disclose on these points even when the disclosure is negative.

Sample size	Samples were chosen based on its data quality after preprocessing (e.g., evaluated using head motion parameters, registration results, seriousness of image distortion due to artifacts and no NAN in partial correlation computation.). Our final sample size is 2696, and the subjects are more densely distributed from birth to 2 years old.
Data exclusions	Subjects for which fMRI motion parameters exceeded the mean Power's FD (absolute sum of motion parameters) of 0.5 mm were excluded. Data that failed visual inspection due to excessive image distortion or failed registration were removed. Samples with NAN during partial correlation computation were removed.
Replication	The results are replicable.
Randomization	Our study does not involve the division of the datasets into multiple groups. Instead, we are tracking how effective connectivity changes with respect to age. Thus, no randomization was required.
Blinding	Our study involved only healthy individuals with a constant experimental condition (resting state fMRI). Thus, no blinding was necessary.

Reporting for specific materials, systems and methods

We require information from authors about some types of materials, experimental systems and methods used in many studies. Here, indicate whether each material, system or method listed is relevant to your study. If you are not sure if a list item applies to your research, read the appropriate section before selecting a response.

Materials & experimental systems

n/a	Involved in the study	
☒	☐	Antibodies
☒	☐	Eukaryotic cell lines
☒	☐	Palaeontology and archaeology
☒	☐	Animals and other organisms
☒	☐	Clinical data
☒	☐	Dual use research of concern
☒	☐	Plants

Methods

n/a	Involved in the study	
☒	☐	ChIP-seq
☒	☐	Flow cytometry
☐	☒	MRI-based neuroimaging

Plants

Seed stocks	N/A
Novel plant genotypes	N/A
Authentication	N/A

Magnetic resonance imaging

Experimental design

Design type	Resting-state functional MRI
Design specifications	Each resting-state fMRI data were acquired in 5 min 47s, and consisted of 420 volumes. Children younger than 3 years of age were imaged during natural sleep while older subjects (> 3 years old) were imaged during passive movie watching during rs-fMRI acquisition. Different phase-encoding directions (anterior-to-posterior (AP), and posterior-to-anterior (PA)) were used, generating at least two rs-fMRI data sets in each visit. For each phase-encoding direction, a single-band reference image (SBref) that has identical voxel resolution, image dimension, phase encoding polarity and echo spacing with the rs-fMRI data was also acquired for motion correction purpose. In addition, a pair of spin echo field maps (FieldMaps) with reversed phase-encode blip (i.e., AP and PA) were also acquired for distortion correction purpose in rs-fMRI data caused by gradient nonlinearity and B0 inhomogeneity. For each subject at each timepoint (some BCP subjects were scanned at multiple ages in staggered-cohort longitudinal study design), resting-state scan sessions (AP/PA) might be repeated for 2-3 times whenever possible, e.g., if the subject is still stationary.
Behavioral performance measures	N/A. Only resting state data were used.

Acquisition

Imaging type(s)	functional MRI
Field strength	3T
Sequence & imaging parameters	The T1w images were scanned with isotropic resolution of 0.8 mm, 320x320 matrix, 256 mm x 256mm FOV, 2.24ms TE, and 2400/1060 ms TR, while T2w images were scanned with isotropic resolution of 0.8 mm, 320 x 320 matrix, 256mm x 256 mm FOV, 564 ms TE, and 3200 ms TR. Rs-fMRI data were collected using single-shot echo-planar imaging (EPI) sequences, with isotropic resolution of 2 mm, 104 x 104 matrix, 208 mm x 208 mm FOV, 37 ms TE, 800/720 ms TR, 52° flip angle, multiband acceleration factor = 8, acquisition time = 5 min 47s, and 420 volumes.
Area of acquisition	whole-brain scans were acquired.

Diffusion MRI ☐ Used ☒ Not used

Preprocessing

Preprocessing software	FSL is used for preprocessing, ANTs is used to register structural data to MNI space, and ICA-AROMA is used for denoising.
Normalization	N/A
Normalization template	N/A
Noise and artifact removal	FSL's mcflirt for motion correction, fsl's topup for EPI distortion correction, and ICA-AROMA for artifacts removal.
Volume censoring	No volume censoring.

Statistical modeling & inference

Model type and settings	Generative Additive Mixture Modeling fitting, using the effective connectivity value as target, and demographic variables (age, scan site, subject ID) as covariates. Age is the covariate of interest, while scan site and subject ID are the random effect.
Effect(s) tested	N/A

Specify type of analysis: ☒ Whole brain ☐ ROI-based ☐ Both

Statistic type for inference (See Eklund et al. 2016): N/A

Correction: N/A

Models & analysis

n/a	Involved in the study
☐	☒ Functional and/or effective connectivity
☒	☐ Graph analysis
☒	☐ Multivariate modeling or predictive analysis

Functional and/or effective connectivity: We used regression dynamic causal modeling (rDCM) to estimate effective connectivity between regions.
